# Chitosan-coated bovine serum albumin nanoparticles for topical tetrandrine delivery in glaucoma: *in vitro* and *in vivo* assessment

**DOI:** 10.1080/10717544.2022.2058648

**Published:** 2022-04-06

**Authors:** Salma El-Sayed Radwan, Riham M. El-Moslemany, Radwa A. Mehanna, Eman H. Thabet, Elsayeda-Zeinab A. Abdelfattah, Amal El-Kamel

**Affiliations:** a Department of Pharmaceutics, Faculty of Pharmacy, Alexandria University, Alexandria, Egypt; b Department of Medical Physiology, Faculty of Medicine, Alexandria University, Alexandria, Egypt; c Center of Excellence for Research in Regenerative Medicine and Applications (CERRMA), Faculty of Medicine, Alexandria University, Alexandria, Egypt; d Medical Research Institute (MRI), Alexandria University, Alexandria, Egypt

**Keywords:** Tetrandrine, chitosan, albumin nanoparticles, glaucoma

## Abstract

Glaucoma is one of the leading causes of blindness. Therapies available suffer from several drawbacks including low bioavailability, repeated administration and poor patient compliance with adverse effects thereafter. In this study, bovine serum albumin nanoparticles (BSA-NPs) coated with chitosan(CS) were developed for the topical delivery of tetrandrine (TET) for glaucoma management. Optimized nanoparticles were prepared by desolvation. pH, BSA, CS and cross-linking agent concentrations effects on BSA-NPs colloidal properties were investigated. CS-BSA-NPs with particle size 237.9 nm and zeta potential 24 mV was selected for further evaluation. EE% exceeded 95% with sustained release profile. In vitro mucoadhesion was evaluated based on changes in viscosity and zeta potential upon incubation with mucin. *Ex vivo* transcorneal permeation was significantly enhanced for CS coated formulation. *In vitro* cell culture studies on corneal stromal fibroblasts revealed NPs biocompatibility with enhanced cellular uptake and improved antioxidant and anti-proliferative properties for the CS-coated formulation. Moreover, BSA-NPs were nonirritant as shown by HET-CAM test. Also, bioavailability in rabbit aqueous humor showed 2-fold increase for CS-TET-BSA-NPs compared to TET with a sustained reduction in intraocular pressure in a rabbit glaucoma model. Overall, results suggest CS-BSA-NPs as a promising platform for topical ocular TET delivery in the management of glaucoma.

## 1. Introduction

Glaucoma is a progressive illness that requires lifelong treatment with medication. Moreover, the poor bioavailability of the currently marketed drugs leads to frequent dosing and poor patient compliance jeopardizing the patients’ vision (Occhiutto et al., 2020). Ninety percent of marketed ophthalmic medications are in the form of eyedrops which is considered a relatively easy and favorable drug delivery route. Nevertheless, ocular drug delivery presents a tremendous challenge owing to the multiple barriers posed by the human eye (Mun et al., 2014). The difficulty of drug diffusion through the multi-layered structure of the eye that includes lipid-rich endothelial and epithelial layers as well as the stroma which is high in water content, in addition to tight junctional complexes in the corneal epithelium, lacrimal turnover, nasolacrimal drainage, blinking reflexes, efflux transporters, drug metabolism by ocular enzymes, and drug binding to or repulsion from conjunctival mucins, tear proteins, and melanin (Lanier et al., 2021) all make efficient ocular drug delivery defying.

This introduces the need for a drug formulation that overcomes ocular barriers and sustains drug release aiming at decreasing dosing frequency. Nanotechnology has recently been investigated as an alternative ocular drug delivery platform with the merits of the “nano” particle size that increases the overall surface area giving it the advantage of easily penetrating biological barriers of the eye (Agarwal et al., 2018).

Tetrandrine (TET) is a bisbenzylisoquinoline alkaloid extracted from the root of Chinese medicinal herb Radix Stephania tetrandra with different pharmacological effects including anti-inflammatory, antiplatelet aggregation, Ca^2+^ channel blocking, immunosuppressive and free radical scavenging actions, so it has been used as an analgesic, diuretic, and anti-inflammatory agent in China (Xiaoyan et al., 2008). Recent studies have shown that TET inhibits the activation of microglia -which may lead to neuronal damage- through the NF-kB and ERK 1/2 signaling pathways, as well as the production of IL1b and TNFa (Li et al., 2020). Furthermore, TET can protect retinal ganglionic cells from ischemic injury *in vitro* and *in vivo* (Li et al., 2014). A study conducted by Huang et al. (2011) to study the effect of 0.3% TET on the IOP of hypertensive rats concluded that topically administered TET 0.3% was effective in lowering IOP and thus, is a promising candidate in the treatment of glaucoma. Nevertheless, owing to TET low aqueous solubility, its ocular bioavailability is limited. Therefore, it is crucial to design a novel ocular drug delivery system that not only improves TET aqueous solubility but also enhances its ocular bioavailability and efficacy. Li et al. (2014) formulated TET-loaded solid lipid NPs, the NPs showed a higher pharmacokinetics profile in aqueous humor. Also, Liu et al. (2016) developed cationic liquid crystalline NPs that increased the pre-ocular residence time and corneal permeation of TET.

Protein NPs have great potential among various ophthalmic carriers such as being safe, nonimmunogenic while enduring prolonged storage conditions. In addition, they are easy to scale up in the manufacturing process as compared to other drug delivery systems (Karimi et al., 2016). Among different types of protein, albumin is an attractive macromolecular carrier for ocular drug delivery because of its natural properties including biodegradability and biocompatibility (Radwan et al., 2021) and it can be considered a potent drug carrier to increase the solubility of poorly soluble compounds (Kim et al., 2019). The presence of functional groups like carboxylic and amino groups on albumin NPs facilitates surface modification via several techniques such as conjugation, coating, or electrostatic adsorption (Elzoghby et al., 2012; Tan and Ho, 2018). Several studies advocated that the albumin-based ocular drug delivery system is multifunctional in nature and capable of expanding both drug residence time and sustaining drug release to deliver the required pharmacological outcomes (Rahul et al., 2021).

Chitosan (CS) is a linear copolymer composed of repeating units of 2-amino-2-deoxy-d-glucan with glycosidic linkages, where the amine groups contribute to CS unique qualities, such as its elevated positive charge and salt-forming properties. Chitosan is a naturally existing polymer of minimum toxicity and is biodegradable (Radwan et al., 2017). In addition, its mucoadhesive properties due to the interaction between the positive amino groups in CS and the negatively charged sialic acid residues in mucin contribute to its extensive study as an ocular drug delivery system (Pahuja et al., 2012; Gharge and Pawar, 2017). Chitosan was suggested to be one of the best polymers to be used in the treatment of glaucoma owing to its mucoadhesive, muco-penetration, and controlled release properties (Kumara et al., 2021).

Chitosan-coated albumin NPs were first formulated by Karimi et al. (Karimi et al., 2013) using the phase separation method and ionic interaction to prepare albumin–CS core–shell NPs for DNA delivery. In preceding research, they were functionalized by MUC-1 to target cancers that overexpress MUC-1. The system was able to encapsulate the hydrophobic drug, paclitaxel, in the albumin inner core, enhancing its permeation to the cells which was attributed to CS positive charge and the active targeting moiety (Esfandyari-Manesh et al., 2016). Albumin NPs were designed as a growth factor carrier by desolvation method and were further stabilized by CS coating by Li et al. (2018). The CS-coated albumin NPs presented favorable stability and sustained release kinetics of the osteogenic protein NELL-1. Moreover, Piazzini et al. (2019) reported the study of CS coated albumin NPs as a promising strategy for the nose to brain drug delivery. However, to our knowledge, this is the first time that CS-coated albumin NPs are studied as an ocular drug delivery platform for topical delivery.

The objective of this study was to formulate and optimize bovine serum albumin nanoparticles for the topical ophthalmic administration of TET for the treatment of glaucoma. CS coating of the NPs is intended to attain increased ocular residence and high transcorneal permeation of the drug thus efficient management of high intraocular pressure. Evaluation of efficacy and safety of the prepared formulation was achieved through *in vitro* techniques, using corneal stromal fibroblasts isolated from adult rats. Moreover, *in vivo* ocular pharmacokinetics and pharmacodynamics were performed in a rabbit model.

## 2. Materials and methods

### 2.1. Materials

Tetrandrine was purchased from ATK chemical (QingPu, Shanghai, China). Bovine albumin fraction V powder was obtained from (Loba Chemie Pvt, Mumbai, India). Low molecular weight Chitosan 50–190 kDa was provided by Sigma Aldrich (St. Louis, MO). Glutaraldehyde was from Al-Gomhureya Chemicals (Cairo, Egypt). Ethanol, Acetonitrile; HPLC grade were from Fisher Scientific (Warrington, UK). All other chemicals and organic solvents were of analytical grade. TAC and MDA kit (Biodiagniostic, Giza, Egypt). Anti-Ki-67 conjugated rabbit monoclonal antibodies Alexa Fluor 488 (IgG, Cell Signaling Technology, Waltham, MA) and the cell surface markers for the corneal fibroblast characterization (CD90, CD105, CD11b, CD45, and CD73) were from (Abcam, Cambridge, UK).

### 2.2. Preparation of BSA-NPs

BSA-NPs were prepared by the desolvation method (Galisteo-González & Molina-Bolívar, 2014). Briefly, BSA (40, 70, and 100 mg) was dissolved in 2 mL 10 mM NaCl and pH was adjusted to 4.9, 6.8, or 8 using 0.1 N NaOH. This was followed by stirring for 10 minutes, then, the desolvating agent; absolute ethanol was added dropwise (1 mL/min) till the formation of an opalescent dispersion. Subsequently, the desolvated BSA-NPs were cross-linked with 10 µl of (2, 4, or 8% v/v) glutaraldehyde, and the reaction was kept under constant mild magnetic stirring for 24 h at room temperature. The resulting colloidal dispersion was centrifuged (Sigma Laboratory Refrigerated Centrifuge, Model 3 K-30; Germany) at 12,000 rpm for 35 min at 4 °C. The supernatant was then removed, and the precipitated BSA-NPs were redispersed in 1 mL PBS (pH 7.4).

TET-loaded BSA-NPs were prepared by dissolving TET (4 mg) in the desolvating agent followed by drop-wise addition to BSA solution.

### 2.3. Preparation of chitosan-coated BSA-NPs

For the preparation of CS-coated BSA-NPs, CS was dissolved overnight in 1% acetic acid then filtered and pH adjusted to 4.5. BSA-NPs prepared as previous were then added dropwise to 1 ml CS solution (0.25, 0.5, 0.75, and 1 mg/mL) and stirred for 15 min.

### 2.4. *In vitro* characterization of BSA-NPs

#### 2.4.1. Colloidal properties and morphology

The mean particle size (PS), polydispersity index (PDI), and zeta potential (ZP) of BSA-NPs were measured by (Nano ZS Series DTS 1060, Malvern Instruments S.A., Worcestershire, UK) at a fixed angle at 25 °C using a 4 mW He–Ne laser at 633 nm. Dispersions were diluted in deionized water and measurements were performed in triplicate.

The morphology of BSA-NPs and CS-BSA-NPs was examined by transmission electron microscopy (TEM) using JEOL, JEM-100 CX Electron Microscope, Tokyo, Japan. Before analysis, the selected dispersions were sprayed onto copper grids without staining. Shots were taken at ×40 K magnification.

#### 2.4.2. Entrapment efficiency

Entrapment efficiency (EE%) was determined after separation of BSA-NPs by centrifugation and measuring the free (un-entrapped) TET concentration in the supernatant using UV spectrophotometer (UV-160A double beam spectrophotometer (Agilent Technologies, Palo Alto, CA)) at 282 nm. TET concentration was calculated using calibration standards. The method was linear in the concentration range 10–60 µg/ml with a determination coefficient of 0.999. Measurements were done in triplicate. The %EE of TET in BSA-NPs was calculated from the difference between initial drug concentration added and free drug concentration in the supernatant. using the following equation:

$$\%\;EE\;=\;\frac{\left(Ci-Cf\right)\;\times \;100}{Ci}$$

where *C_i_* is the initial drug content and *C_f_* is the free drug in the supernatant.

#### 2.4.3. *In vitro* drug release study

TET release from TET-BSA-NPs and CS-TET-BSA-NPs compared to TET suspension in phosphate buffer saline was studied using the dialysis method. VISKING^®^ dialysis tubing MWCO 12,000–14,000 was used. A constant volume (1 ml) of NP formulation dispersion in PBS equivalent to 4 mg TET was placed in presoaked dialysis bags and suspended fully immersed in the release medium (PBS pH 7.4) maintaining sink conditions (Liu et al., 2016). TET release was then assessed in a thermostatically controlled shaking water bath (Köttermann GmbH, Hänigsen, Germany) at 37 °C and 100 rpm. At preplanned intervals (0.25, 0.5, 1, 2, 3, 4, 5, 6, 24, and 48 h), samples were collected and replaced with an equal volume of fresh release medium. The concentration of the drug was determined spectrophotometrically at 282 nm. The % cumulative TET released was calculated in triplicate.

The kinetics of drug release from NPs dispersions in comparison with TET suspension were assessed using model-dependent methods (Costa & Sousa Lobo, 2001) calculated by the Excel add-in; DDsolver (Zhang et al., 2010).

#### 2.4.4. *In vitro* mucoadhesion studies

*In vitro* mucoadhesion of TET-BSA-NP and CS-TET-BSA-NPs was assessed as reported (Radwan et al., 2021) by measuring changes in ZP and viscosity following incubation of the formulation with 0.1%w/v mucin at pH 7.4. To ensure complete dispersion, mucin was first hydrated with distilled water, followed by gentle stirring at room temperature. Both ZP and viscosity measurements were done in triplicate. NPs control samples were prepared by replacing mucin with distilled water, whereas the mucin control sample was prepared by two-fold dilution of hydrated mucin with distilled water.

The interaction of NP formulations with mucin was determined by measuring the change in ZP compared to NPs and mucin controls following incubation of NPs/mucin mixtures in the ratio 1:1 for 2 h at room temperature. ZP was measured as previously described.

The viscosity was measured at room temperature after incubation of tested formulations with mucin in a 1:1 ratio for 30 min using cone and plate viscometer (DV2T, Brookfield, Middleboro, MA) at 10 rpm using spindle 40.

### 2.5. *Ex vivo* corneal permeability

Rabbit corneas from healthy adult white male New Zealand rabbits were freshly procured to assess *ex vivo* corneal permeation of TET using the modified Franz diffusion method. Corneas with 2-3 mm sclera were mounted on Franz diffusion cells (0.5 cm^2^) between donor and receiver compartments with the endothelial side facing the latter and set at 37 ± 0.5 °C at 50 rpm (Parra et al., 2015). The receptor compartment comprised freshly prepared PBS pH 7.4 (2 ± 0.2 ml) as the diffusion medium. TET-BSA-NPs, CS-TET-BSA-NPs, and TET suspension in PBS (0.5 ml containing 2 mg TET) were placed on the rabbit cornea representing the donor side. Half-milliliter samples were withdrawn at various time points (0.25, 0.5, 1, 1.5, 2, 3, 4, 5, and 6 h) with replacement and were analyzed using UV spectrophotometry, after suitable dilution. The apparent corneal permeability coefficient (*P*_app_) was calculated from the following Equation (Liu et al., 2016):

$$P_{\mathrm{app}}=\;\frac{\Delta Q}{\Delta t.C_{o}.A.60}$$

where Δ*Q*/Δ*t* is the slope of the linear portion of the cumulative permeated amount-versus-time plot, *C*_0_ is the initial TET concentration in the donor compartment, *A* is the exposed corneal surface area (0.5 cm^2^), and 60 was used for conversion from minutes to seconds.

### 2.6. Cell culture studies

#### 2.6.1. Preparation of sterile BSA-NPs

TET-BSA-NPS and CS-TET-BSA-NPs dispersions were prepared under aseptic conditions. Albumin, GA, and drug alcoholic solutions were sterilized by filtration through 0.22 µm sterile Millipore filters (Millipore Corp., Santa Carla, CA), then the NPs preparation procedure was executed under a vertical laminar flow hood as previously mentioned. The sterile NP suspensions were then cultured on agar plates and assessed after 24 h for bacterial colonies. All cell culture studies were conducted in the Center of Excellence For Research in Regenerative Medicine and its applications CERRMA, Faculty of Medicine, Alexandria University.

#### 2.6.2. Isolation and culture of corneal stromal fibroblasts

Isolation of corneal stromal fibroblasts from adult rats was done using a corneal explant protocol (Nagymihaly & Moe, 2020). Briefly the adult male rats were sacrificed, and the eyeballs were dissected out in a petri dish in PBS. The corneal-limbal junction was identified and the corneal button was carefully dissected out using sharp scissors. The corneal button was placed in dispase II solution consisting of 15 mg/ml dispase II, 100 mM Sorbitol, and 1% penicillin/streptomycin in DMEM high glucose basal medium, on ice. The dispase was washed out twice with PBS. In a culture dish, the corneal tissue was cut into 2 × 2 mm pieces and plated in a 12- well plate. Culture medium (DMEM high glucose, 10% FBS, 1% penicillin/streptomycin, Lonza, Basel, Switzerland) was added to the plate to cover the tissue explants (200–250 μL) and then incubated in 5% CO2 and 37 °C. After the first day, the culture medium was added up to 1–1.5 mL to the wells with the corneal explants. The media were refreshed every 3–4 days. Once spindle adherent cells from tissue explants reached 80-90% confluency, they were trypsinized using 0.25% trypsin EDTA (Lonza, Basel, Switzerland) and expanded in larger T75 cm^2^ flasks (Nagymihaly & Moe, 2020).

#### 2.6.3. Characterization of corneal fibroblasts

##### 2.6.3.1. Morphology

Daily morphology of the explants and cultured cells were monitored using inverted bright field microscopy (Olympus CKX41SF, Tokyo, Japan).

##### 2.6.3.2. Immunophenotyping

Once confluent, cells were trypsinized and characterized using fluorescent-labeled monoclonal antibodies (mAb) for CD 44, CD90, CD105, CD 11 b, CD 45, and CD 73 markers. Following trypsinization with 0.25% trypsin-EDTA solution, cells were washed with PBS, and incubated at room temperature in the dark for 30 min with monoclonal phycoerythrin (PE)-conjugated antibody for CD105, monoclonal Allophycocyanin-conjugated antibody for CD73 (Abcam, Cambridge, UK), monoclonal PE-conjugated antibody for CD11b, and monoclonal PE-conjugated antibody specific for CD45 and CD 90 all at dilutions 1:200. Cells were washed with PBS and resuspended in 500 ml FACS buffer (1% BSA in PBS). The fluorescent intensity of the labeled cells was analyzed using Becton Dickinson, FACS caliber flow cytometer operated with Cell Quest software (Becton Dickinson, Franklin Lakes, NJ) (Young, 2001).

#### 2.6.4. MTT cytotoxicity assay

Corneal stromal fibroblasts were grown as monolayer cultures in the culture media mentioned above. Once confluent, cells were trypsinized and plated into two 96-well plates with a uniform seeding density of 7 × 10^3^ cells/well and incubated with different formulations for 24 h. The following day the medium was replaced with medium containing different concentrations of the TET suspension and TET loaded BSA-NPs and CS-BSA-NPs ranging from 0.002 µg/mL to 20 µg/mL and left for 48 h at 37 °C and 5% CO_2_. After 48 h the media from the corresponding plates were aspirated, replaced by MTT solution, and incubated for 4 h. Lastly, the formazan blue crystals were dissolved in DMSO and the absorbance at 570 nm was measured by ELISA well-plate reader (Tecan, Infinite F50, Männedorf, Switzerland). The values obtained were compared with the control that was regarded as 100% living cells (Fellows & O'Donovan, 2007).

#### 2.6.5. Cellular uptake study

Fluorescently labeled BSA-NPs and CS-BSA-NPs were prepared using coumarin 6 (C6). A similar desolvation procedure was adopted with the addition of 50 μg of the dye to ethanol. The incorporated dye offers a sensitive method to quantitate the intracellular uptake and retention (Parveen & Sahoo, 2011).

Corneal stromal fibroblasts were seeded in 6-well plates at an initial density of 3 × 10^5^ cells/well on coverslips. Once confluent, C6-loaded BSA-NPs both uncoated and coated with CS and the free coumarin dye were added to the corresponding wells and incubated at 37 °C and 5% CO_2_ for 48 h. Then the cells were washed with PBS, fixed with 4% PFA at room temperature for 10 min in the dark ad washed again before mounting. The cells were observed using laser scanning confocal microscopy (×63, oil objectives Leica microsystems, DMi8, Germany) and the fluorescent signal of the conjugated formulations were analyzed and compared with the control and the freed drug. The uptake study was conducted in duplicates.

#### 2.6.6. Proliferation marker (Ki-67)

A sample of the confluent cells exposed to different drug formulations (TET, TET-BSA, and CS-TET-BSA) was trypsinized and turned into single-cell suspension for intracellular staining with the anti-Ki67 antibody. First, cells were fixed with 4% PFA at room temperature for 10 min followed by permeabilization for 30 mins using 1% triton-x. Blocking was done using 2% BSA for an additional 30 min to avoid nonspecific binding. The cells were finally stained with specific Ki-67 conjugated MoAb and incubated at 4 °C for 60 min in the dark. The fluorescent intensity of the labeled cells was analyzed using Becton Dickinson, FACS caliber flow cytometer operated with Cell Quest software (Becton Dickinson, Franklin Lakes, NJ) (Marcondes et al., 2018).

#### 2.6.7. Evaluation of oxidative stress

The conditioned media of cultured fibroblasts exposed to different drug formulations and from the controls were collected and used to measure total antioxidant capacity (TAC) and Lipid peroxide (malondialdehyde, MDA).

##### 2.6.7.1. Total antioxidant capacity assay (TAC)

TAC was assessed using a commercially available TAC kit and was conducted according to the manufacturer’s instructions. Briefly, the antioxidants present in cell culture media react with the exogenously provided hydrogen peroxide (H_2_O_2_). The residual H_2_O_2_ is detected using colorimetry and the TAC was calculated by subtracting the absorbance of the samples from the blank and was expressed as (mM/L). The experiment was conducted in triplicates (Young, 2001)

##### 2.6.7.2. Lipid peroxide (malondialdehyde) assay

A sample of the conditioned media was used to measure the lipid peroxidation products by a commercial MDA kit using thiobarbituric acid (TBA) that reacts with MDA in an acidic medium at 95 °C for 30 min to form a colored complex. Absorbance was measured spectrophotometrically at 534 nm. MDA standard was used to create a standard curve and the readings of the samples were plotted. The level of lipid peroxidation was calculated and expressed as nmol MDA per ml. The experiment was conducted in triplicates (Satoh & Butler, 1978).

### 2.7. Ocular tolerance test – HET-CAM

The modified hen’s egg chorioallantoic membrane (HET-CAM) test (Khalaf et al., 2022) was carried out to evaluate the ocular tolerability of TET suspension, TET-BSA-NPs, and CS-TET-BSA-NPs. The potential irritancy of compounds may be detected by observing adverse changes that occur in the CAM of the egg after exposure to test chemicals. Briefly, fertilized hens’ eggs were maintained in the incubator for 10 days at a controlled temperature (37.8 °C) and humidity (50–60%). Following incubation, the shell was cut a little above the marked line of the CAM and this section of the shell was removed. The inner membrane directly in contact with the CAM was dampened with 1 ml of 0.9% saline solution. The inner membrane was then removed with great care to avoid harming blood vessels, then it was possible to see the CAM below. A volume of 0.5 mL of the studied formulation was added directly by pipette onto the CAM. Hemorrhaging and/or coagulation after a 5-min period following the application of the test solution were written down and evaluated according to a scoring system, and any effect that stood out as compared with the controls: saline (negative) and 0.1 sodium hydroxide (positive) solutions. Samples were tested in triplicate.

The ocular irritation index (OII) was calculated by the following equation according to the scoring system described by Alvarado et al. (2015):

$$\mathrm{OII}=\frac{(301-h)\times 5}{300}+\frac{(301-l)\times 7}{300}+\frac{(301-c)\times 9}{300}$$

where *h*, *l,* and *c* are times of the beginning of hemorrhage, lysis, and coagulation, respectively. The following classification was used: *OII* ≤ 0.9 slightly irritating; 0.9 < *OII* ≤ 4.9 moderately irritating, 4.9 < *OII* ≤ 8.9 irritating, and 8.9 < OII ≤21 severely irritating.

### 2.8. *In vivo* characterization

#### 2.8.1. Animals

Male New Zealand rabbits, weighing 1.5–2 kg, were provided by the animal facility of the Faculty of Agriculture, Alexandria University. Rabbits were housed at ambient temperature with free access to food and water. Experiments were performed in accordance with the guidelines of the Institutional Animal Care and Use Committee, Faculty of Pharmacy, Alexandria University ((Approval number: AU-062021-2-104).

#### 2.8.2. Aqueous humor pharmacokinetics

##### 2.8.2.1. Study design

A total of 9 rabbits were used for the study of the aqueous humor pharmacokinetic profile of TET suspension, TET-BSA-NPs, and CS-TET-BSA-NPs (*n* = 3). A dose equivalent to 450 μg TET was instilled in each eye (three 50 μL drops at 5 min intervals). Aqueous humor from the dosed eyes of the rabbit was withdrawn at (0.5, 1, 2, 4, 6, 8, and 12 h) with the time of instilling the last formulation drop taken as zero time. The samples were collected and processed as described (Singh et al., 2019).

##### 2.8.2.2 Aqueous humor sampling

After anesthesia using 35 mg/kg ketamine and 5 mg/kg xylazine (Satheshkumar, 2005), a 30-gauge needle was inserted above the corneoscleral junction traversing through the center of the anterior chamber to withdraw aqueous humor. At each time interval, 100 μL aqueous humor was withdrawn from each eye. The aqueous humor was collected in labeled polypropylene tubes and stored at −80 °C until further analysis.

##### 2.8.2.3. Tetrandrine quantification in aqueous humor

Tetrandrine was analyzed in aqueous humor samples using an HPLC-MS method. Aqueous humor samples were vortex-mixed with 400 μL acetonitrile for 1 min and centrifuged at 15,000 rpm to separate precipitated proteins. The supernatant was filtered through a 0.22-μm syringe filter prior to analysis. Separation was carried out on a reversed-phase C18 column (ZORBAX Eclipse Plus C18 column, 4.6 × 150 mm, 5 μm). An isocratic eluent consisting of methanol: acetonitrile, 3:2 (mobile phase A) and 0.1% aqueous formic acid (mobile phase B) in a ratio of 50:50 v/v was used. The injection volume was 20 μL and the flow rate was adjusted to 0.4 mL/min. Elution was carried out at room temperature. The LC-MS system used was Shimadzu^®^ UFLC series (Shimadzu Corporation, Kyoto, Japan) (control unit CBM-20A, 2 pumps LC-20AD, thermally controlled autosampler SIL-20AC, degasser DGU-20A, column oven CTO-20AC) coupled with UV detector SPD-20A and MS detector LCMS-2020 with single quadrupole system and two ion sources ESI or APCI. The data was processed using LabSolutions LCMS^®^ Software (release 5.42 SP4 for LCMS-2020). Mass range: *m*/*z* 10–2000, sensitivity: ESI Reserpine 1 pg S/N (RMS)>100, APCI Reserpine 1 pg S/N (RMS)>100, Scan rate: up to 15,000 amu/s.

TET concentration was calculated using calibration standards for peak area in spiked aqueous humor. The method was linear in the concentration range 5–100 µg/mL with a determination coefficient of 0.999 and inter-and intra-day precision less than 4.0%. Measurements were done in triplicate.

The concentrations versus time data were analyzed based on a non-compartmental pharmacokinetic model using Excel pharmacokinetic solver add-in (Zhang et al., 2010). The main pharmacokinetic parameters for all groups were calculated. Results were expressed as mean ± standard deviation (SD) (*n* = 3).

#### 2.8.3. *In vivo* antiglaucoma activity

Ocular hypertension was induced by topical application of 50 µL 1% cortisone 2 times daily for 7 days with a follow-up of intraocular pressure (IOP) using Schiotz tonometer (Riester GmbH & Co. KG, Jungingen, Germany). Rabbits were then randomly divided into three groups: TET suspension, TET-BSA-NPs, and CS-TET-BSA-NPs (*n* = 3). For all animals, the right eye served as control receiving only saline 0.9% while the left eye received treatment equivalent to 150 μg TET. The selected dose was chosen according to study carried out by Huang et al. (2011). IOP was measured directly before drug administration (zero time) and 0.5, 1, 2, 3, 4, 6, 8, and 12 h after instillation. IOP was measured three times at each time interval and the means were recorded. Local anesthetic eye drops were used prior to every IOP measurement. The activity was confirmed by noticing a bulge formation at the site of injection. The % change in IOP (ΔIOP) was expressed as follows:

$$\Delta \mathrm{IOP}=\frac{{\mathrm{IOP}}_{\mathrm{zero}\;\mathrm{time}}-{\mathrm{IOP}}_{\mathrm{after}\;\mathrm{treatment}\;}}{{\mathrm{IOP}}_{\mathrm{zero}\;\mathrm{time}}}\times 100$$

### 2.9. Statistical analysis

Data were analyzed by one-way ANOVA test followed by pairwise Tukey test using SPSS 20.0 (SPSS Inc., Chicago, IL). Differences at *p* values ≤ .05 were considered statistically significant.

## 3. Results and discussion

### 3.1. *In vitro* characterization of BSA-NPs

#### 3.1.1. Colloidal properties

BSA has recently gained attention in the pharmaceutical industry owing to its abundance, relatively low cost, and ease of purification. The most famous method of BSA preparation is the well-established desolvation technique (Marty et al., 1978); dehydration of the aqueous solution of BSA is carried out by the addition of an organic solvent such as acetone or ethanol. In this study, ethanol was used as the desolvating agent for its ability to solubilize the nonpolar groups in BSA disrupting its secondary structure with lesser volume compared to other solvents (Mohammad-Beigi et al., 2016). Moreover, it’s less toxic when compared to other solvents. Following desolvation, cross-linking with glutaraldehyde stabilizes the NPs which is kept under mild stirring conditions overnight to ensure sufficient crosslinking of the amino groups of BSA (An & Zhang, 2017). In the current experiment, different variables were investigated for their effect on PS, PDI, and ZP of BSA-NPs: pH, BSA concentration, glutaraldehyde concentration, and chitosan concentration (Table 1).

**Table 1.** Effect of pH, BSA concentration, glutaraldehyde concentration, and chitosan concentration on the particle size, polydispersity index, and zeta potential of the prepared BSA-NPs (*n* = 3).

Code	pH	BSA (mg/mL)	GA (mg%)	CS (mg/mL)	PS (nm)	PDI	ZP (mV)
B1	6.8	50	8	–	373 ± 56	0.59 ± 0.06	−24 ± 2.5
B2	6.8	35	8	–	309 ± 17	0.42 ± 0.02	−30 ± 0.2
B3	6.8	20	8	–	176 ± 2	0.08 ± 0.02	−37 ± 1.3
B4	4.9	20	8	–	1127 ± 146	0.19 ± 0.08	−26 ± 0.9
B5	8	20	8	–	155 ± 1	0.24 ± 0.01	−43 ± 0.8
B6	8	20	4	–	119 ± 0.2	0.09 ± 0.01	−52 ± 1.1
B7	8	20	2	–	122 ± 1	0.08 ± 0.02	−49 ± 0.5
B8	8	20	0	–	137 ± 1	0.11 ± 0.01	−43 ± 0.2
TET-BSA-NPs	8	20	4	–	110.9 ± 11	0.13 ± 0.02	−29 ± 0.5
CS-TET-BSA-NP1	8	20	4	0.25	2293 ± 1724	0.69 ± 0.45	−6 ± 0.2
CS-TET-BSA-NP2	8	20	4	0.5	1567 ± 616	0.28 ± 0.09	7 ± 0.5
CS-TET-BSA-NP3	8	20	4	0.75	955 ± 16	0.31 ± 0.06	15 ± 0.1
CS-TET-BSA-NP4	8	20	4	1	237.9 ± 3.61	0.42 ± 0.01	24 ± 1.1

BSA: bovine serum albumin; GA: glutaraldehyde; CS: chitosan; PS: particle size; PDI: polydispersity index; ZP: zeta potential.

Increasing BSA concentration from 20 to 50 mg/ml at a constant pH 6.8 and 8 mg/mL GA showed a significant increase in both PS and PDI with a significant decrease in ZP (*p* ≤ .01) (Table 1). These findings are in agreement with the findings of Wang et al. (Wang et al., 2021) and Galisteo-González et al. (Galisteo-González & Molina-Bolívar, 2014). This decrease in PS and PDI could be explained by the theory of nucleation; as the concentration of BSA increases, viscosity increases leading to a decline in transport frequency of protein between water and organic solvent which results in slower nucleation rates and larger PS (Galisteo-González & Molina-Bolívar, 2014). BSA concentration of 20 mg/mL (B3) exhibited the smallest PS and polydispersity (PDI) with the highest ZP (176 ± 2 nm, 0.08 ± 0.02, and −37 ± 1.3 mV). Accordingly, BSA (20 mg/mL) was chosen for further optimization of BSA-NPs.

The effect of pH on the colloidal properties of BSA-NPs was investigated. Significant reduction (*p* ≤ .05) in mean particle diameter (Table 1) was observed with increasing pH of the BSA solution from 4.9 to 8 (1127 ± 146 nm, 176 ± 2 nm, and 155 ± 1 nm for pH 4.9, 6.8, and 8, respectively) while maintaining a high ZP indicating high stability of NPs formed. At pH values higher than the iso-electric point of BSA (pH >5), the net charge of BSA NPs is highly negative resulting in electrostatic repulsion and decline in coagulation of molecules and hence smaller NPs (Galisteo-González & Molina-Bolívar, 2014).

Further, the effect of GA concentration (0, 2, 4, and 8%) as a cross-linker added after the desolvation process was studied at pH 8. GA acts by binding the amino moieties in lysine residues of different BSA molecules in the particle matrix (Galisteo-González & Molina-Bolívar, 2014). Findings suggested a significant decline in both PS and PDI (*p* ≤ .01) as % GA decreased from 8 to 4% (155 ± 1 nm and 0.24 ± 0.01 to 119 ± 0.2 nm and 0.09 ± 0.01, respectively), on the other hand, ZP increased from (−43 ± 0.8 to −52 ± 1 mV, respectively). According to the aforementioned results, formulation B6 prepared using 20 mg/mL, BSA, at pH 8 and 4%, GA was selected for drug loading.

The effect of TET loading into BSA-NPs (B6) at TET concentration 0.4% w/v of the final dispersion volume on colloidal properties was investigated. A slight reduction in PS with a significant decrease in ZP (*p* ≤ .05) was observed (-52 ± 1.1 to −29 ± 0.5 mV for blank and loaded BSA-NPs, respectively) this is because TET is a positive alkaloid (Lu et al., 2015) that might have interacted with BSA-NPs negative charge. Reduction in particle size following drug loading into BSA-NP was similarly reported for apatinib (Radwan et al., 2021).

Different CS concentrations were studied for the preparation of CS-TET-BSA-NPs with good colloidal properties suitable for ocular administration. It was observed that as the concentration of CS increased from (0.25–1 mg/mL) the PS decreased significantly (*p* ≤ 0.01) from 2293 ± 1724 nm to 237.9 ± 3.61 nm. On the contrary, the ZP increased until it reached +24 ± 1.2 mV (CS-TET-BSA-NP4). The increased positive ZP was possibly ascribed to the amine groups in the CS structure (Wang et al., 2013), proposing that CS is effectively coated onto the BSA nanoparticles surfaces (Li et al., 2018). This relatively high ZP is expected to enhance the stability of the NP dispersion since it prevents NPs agglomeration (Radwan et al., 2017). Consequently, CS-TET-BSA-NP4 was selected for further studies.

#### 3.1.2. Morphology

**Figure 1.**
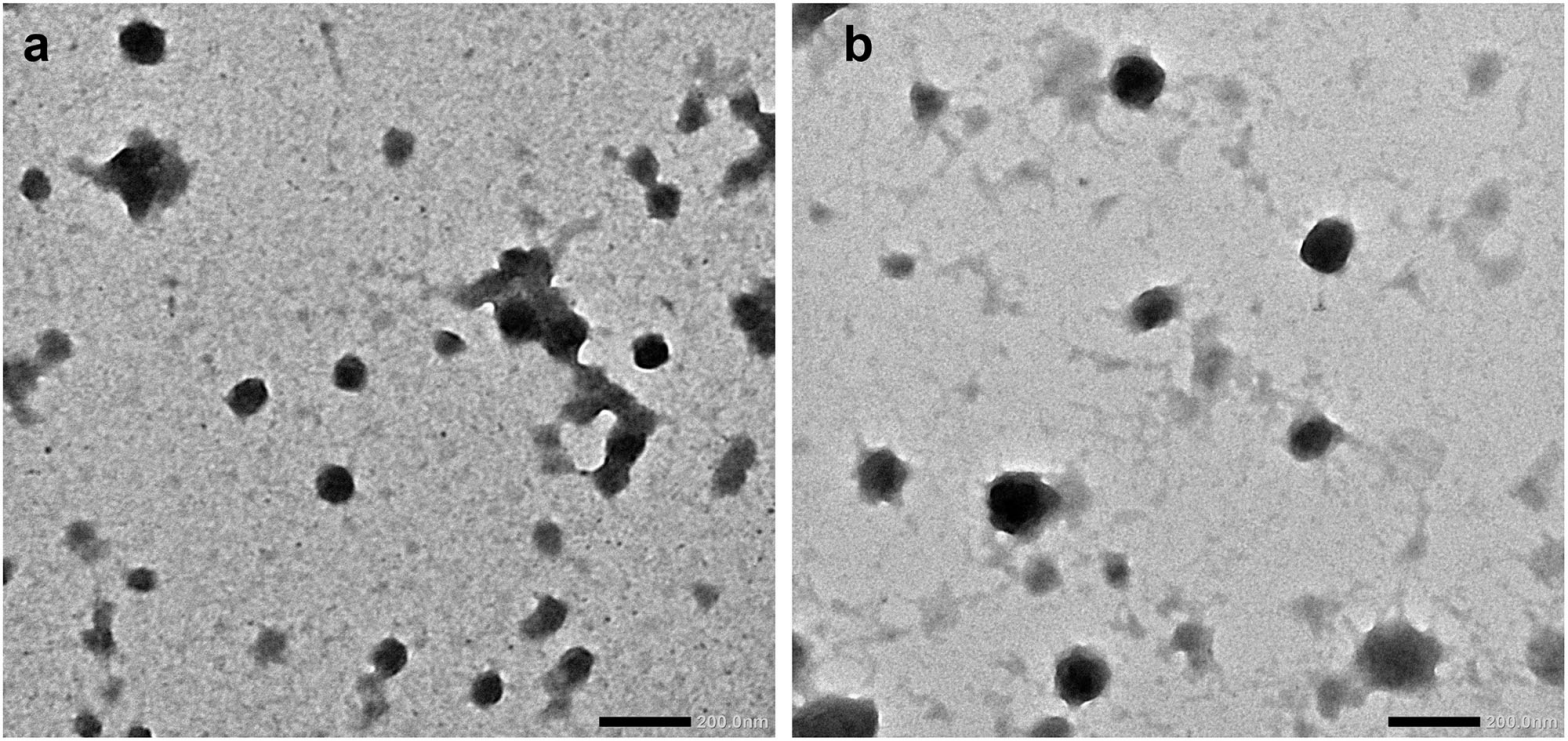
TEM images of (a) BSA-NPs and (b) CS-coated BSA-NPs. Magnification ×40K, scale bar represents 200 nm.

The morphology of TET-BSA-NP and CS-TET-BSA-NPs was investigated using TEM imaging (Figure 1). Images displayed solid spheres with smooth surface and narrow distribution of the NPs. The size enlargement was observed following CS coating.

#### 3.1.3. Entrapment efficiency

The results of spectrophotometric analysis of unentrapped TET showed that it was successfully entrapped in the system with an EE% 98.47 ± 0.9%. CS coating resulted in a slight insignificant reduction in EE% (*p* > .05). The effect increased slightly with increasing CS concentration (97.7 ± 0.5, 96.4 ± 2.6, 96 ± 0.1 and 95.8 ± 0.3% for 0.25, 0.5, 0.75 and 1 mg/mL, respectively).

#### 3.1.4. *In vitro* drug release

**Figure 2.**
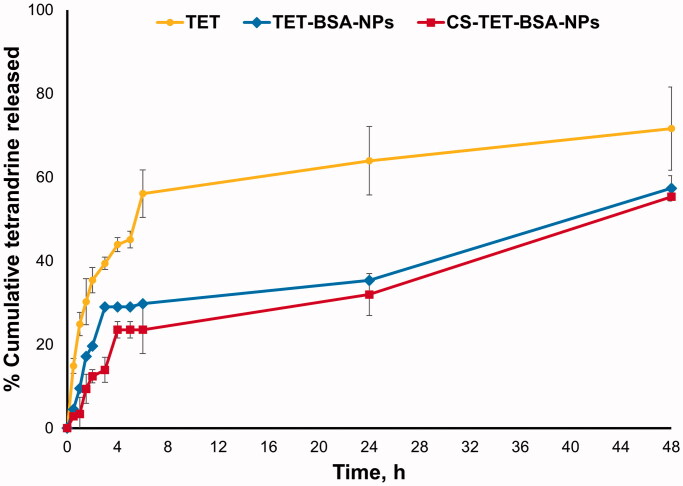
*In vitro* release profile of TET from TET suspension, TET-BSA-NPs and CS-TET-BSA-NPs in PBS pH 7.4 (*n* = 3).

*In vitro* release profiles of TET from TET suspension, TET-BSA-NPs and CS-TET-BSA-NPs are shown in Figure 2. TET suspension showed 35.6 ± 0.65% release after 2 h and reached 60 ± 0.23% after 6 h. By the end of the experiment, 71.63 ± 0.59% TET was released. Li et al. (2020) reported that as TET is a dibenzyl isoquinoline alkaloid, its solubility is affected by the solution’s pH. TET solubility was lower at pH 7.4 compared to pH 6. This possibly explains why TET was not 100% released in the current experiment. Nevertheless, PBS pH 7.4 was chosen for the comparative *in vitro* release study as it mimics the ocular pH. TET loading into BSA-NPs resulted in a sustained drug release with a highly significant decrease in drug burst (19.65 ± 0.23% at 2 h) compared to TET suspension (35.6 ± 0.65%) reaching a maximum of 57.3% after 48 h. CS coating of BSA-NPs resulted in a further significant reduction in drug release with only 12.38 ± 0.02% burst. Despite the significantly slower release (*p* ≤ .05) of TET from CS-coated formulation compared to the uncoated one in early time samples; up to 6 h, this difference disappeared later on probably due to the hydration and swelling of CS coat by time as previously reported (Raj et al., 2018; Piazzini et al., 2019).

Release kinetics were determined by plotting the release data over the study period according to first-order, Higuchi diffusion, Korsmeyer–Peppas, Hixson–Crowell, and Baker–Lonsdale equations. The highest correlation coefficient (*r*^2^) was used as a statistical parameter to designate data best fit. The release of TET from TET suspension, TET-BSA-NPs, and CS-TET-BSA-NPs showed the highest correlation coefficient with the Korsmeyer–Peppas model (0.94, 0.90, and 0.93, respectively) with a Fickian release exponent for TET suspension and TET-BSA-NPs (*n* = 0.25 and 0.33, respectively), suggesting drug release primarily via diffusion. Regarding CS-TET-BSA-NPs, the value of the diffusion exponent (*n* = 0.5), indicated that drug release is anomalous, non-Fickian diffusion combining drug diffusion and matrix erosion due to swelling of polymer chains (Öztürk et al., 2020).

#### 3.1.5. Mucoadhesive properties

**Figure 3.**
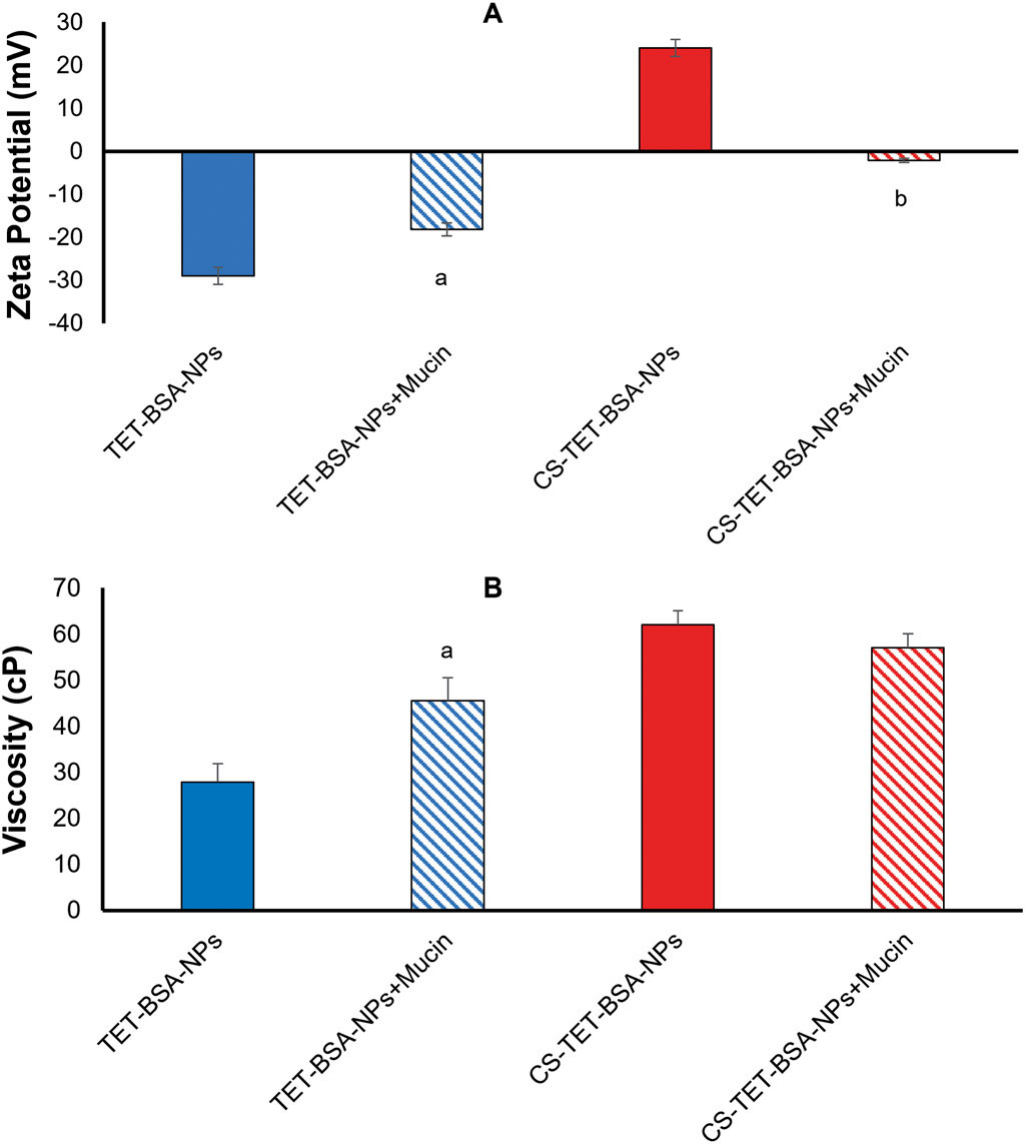
Zeta potential (A) and viscosity (B) of TET-BSA-NPs and CS-TET-BSA-NPs before and after incubation with mucin as an indicator of mucoadhesion (*n* = 3). ^a^*p* < .05 versus TET-BSA-NPs, ^b^*p* < 0.01 versus CS-TET-BSA-NPs.

Strong mucoadhesion proposes a closer contact with the absorption site, thus effective absorption following topical ocular administration (Luo et al., 2015). Mucoadhesive properties were determined by ZP variation and viscosity measurements upon incubation with mucin to predict the interaction of TET-BSA-NPs and CS-TET-BSA-NPs with the corneal mucous and hence prolonged retention (Figure 3).

Mucin 0.1% exhibited a ZP of −12 ± 1 mV. Following incubation of TET-BSA-NPs with mucin, the negative charge decreased significantly (*p* ≤ .05) from −29 ± 0.5 to −18.2 ± 0.5 mV indicating an interaction with mucin (Figure 3(A)). A further indicator of NP/mucin interaction was the significant increase in viscosity (*p* ≤ .05) following incubation of TET-BSA-NPs with mucin (27.9 ± 5.4 to 45.5 ± 0.4 cP before and after incubation, respectively) (Figure 3(B)).

CS-coated TET-BSA-NPs, ZP (+ 24 ± 1.1 mV), showed neutralization of positive charge with a shift to negative ZP (-2.14 ± 1 mV) following incubation (Figure 3(A)) evidencing a highly significant level (*p* ≤ .01) of ionic interaction with mucin (Piazzini et al., 2019). On the contrary, no significant change (*p* > .05) in viscosity was observed (62.02 ± 2.9 and 57.1 ± 2.5 cP before and after incubation, respectively). The mucoadhesiveness of CS is well established in the literature (Rossi et al., 2000; Cordeiro et al., 2021). Low-molecular weight CS coating was previously reported to possess chain flexibility for interacting with mucin, enabling the NP to penetrate into mucin branching sugars (Silva et al., 2017). Moreover, interactions between amino groups on CS and active groups on the sugar lateral chains of mucin contribute to stronger mucoadhesivity (Thongborisute & Takeuchi, 2008). The insignificant change in viscosity observed in the current study could be attributed to the low concentration of mucin used in the study and the relatively high viscosity of the CS-coated formulation. It is worth noting that, Rossi et al. (2001) reported a decline in viscosity which was explained to be a consequence of mucin adsorption onto chitosan.

### 3.2. Transcorneal permeation

**Figure 4.**
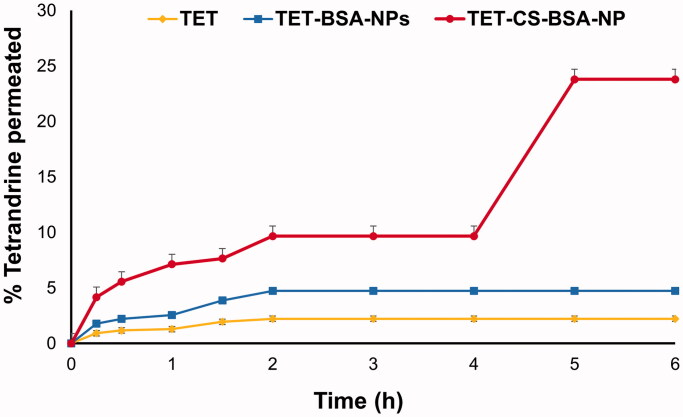
*Ex vivo* corneal permeation profile of tetrandrine from TET-BSA-NPs and CS-TET-BSA-NPs compared to TET suspension (mean ± SD, *n* = 3).

Table 2 and Figure 4 show the cumulative amount of TET permeated into the receiver medium over 6 h and the apparent permeability coefficient (*P*_app_). TET suspension showed minimal permeation (2.21 ± 0.7% after 6 h). A significant improvement (*p* ≤ .05) in % TET permeated (4.72 ± 0.29%) and 2.3-fold increase in *P*_app_ were observed for TET-BSA-NPs. Moreover, CS-TET-BSA-NPs exhibited further significant enhancement (*p* ≤ .01) in % TET permeated (11- and 5-folds increase compared to TET suspension and TET-BSA-NPs, respectively) with 4- and 1.7-folds increase in *P*_app,_ respectively (Table 2).

**Table 2.** *Ex vivo* transcorneal permeation of TET suspension, TET-BSA-NPS, and CS-BSA-NPs expressed as total amount permeated, percentage permeation, and apparent permeability coefficient (*P*_app_) using rabbit cornea (*n* = 3).

Formula	Total amount of TET permeated in 6 h (µg/mL)	% Permeation	Apparent permeability coefficient (*P*_app_) (cm/s)
TET suspension	44.2 ± 0.02	2.21 ± 0.7	1.2 × 10^−5^ ± 0.32
TET-BSA-NPs	94.4 ± 0.1	4.72 ± 0.29	2.8 × 10^−5^ ± 0.5
CS-TET-BSA-NPs	475.8 ± 0.13	23.79 ± 0.9	4.76 × 10^−5^ ± 0.07

The ability of the ocular formulation to permeate the corneal barrier depends on various factors like chemical nature, charge, particle size, and conformation. Nanoparticulate formulations offer several advantages such as small particle size and large surface area and hence, enhance corneal penetration by penetrating the endothelial cell gap (Zhang et al., 2021). Positively charged NPs such as CS-coated NPs enhance permeation by opening the tight junctions located in the corneal epithelial cells (Mittal & Kaur, 2019). Higher permeation of TET from CS-coated formulation might also be attributed to increased retention time at the corneal surface due to the mucoadhesive nature of the cationic polymer CS and the ionic interaction of its protonated amino group with the negatively charged corneal mucin. Also, the endocytic uptake of CS-TET-BSA-NPs as well as improved transport of released drug via paracellular route due to widening of tight junction in the presence of CS as previously reported (Tara & Abdullah, 2016). The *ex vivo* trancscorneal permeation results clearly demonstrated the superiority of CS coated TET-BSA-NPs regarding topical ophthalmic delivery of TET.

### 3.3. *In vitro* cell culture studies

Working under aseptic conditions along with terminal sterilization was used in this study (Rodrigues et al., 2020). All formulations were found to be sterile which was verified by the absence of bacterial colonies after the culture onto agar plates for 24 h.

#### 3.3.1. Corneal explants and fibroblasts characterization

**Figure 5.**
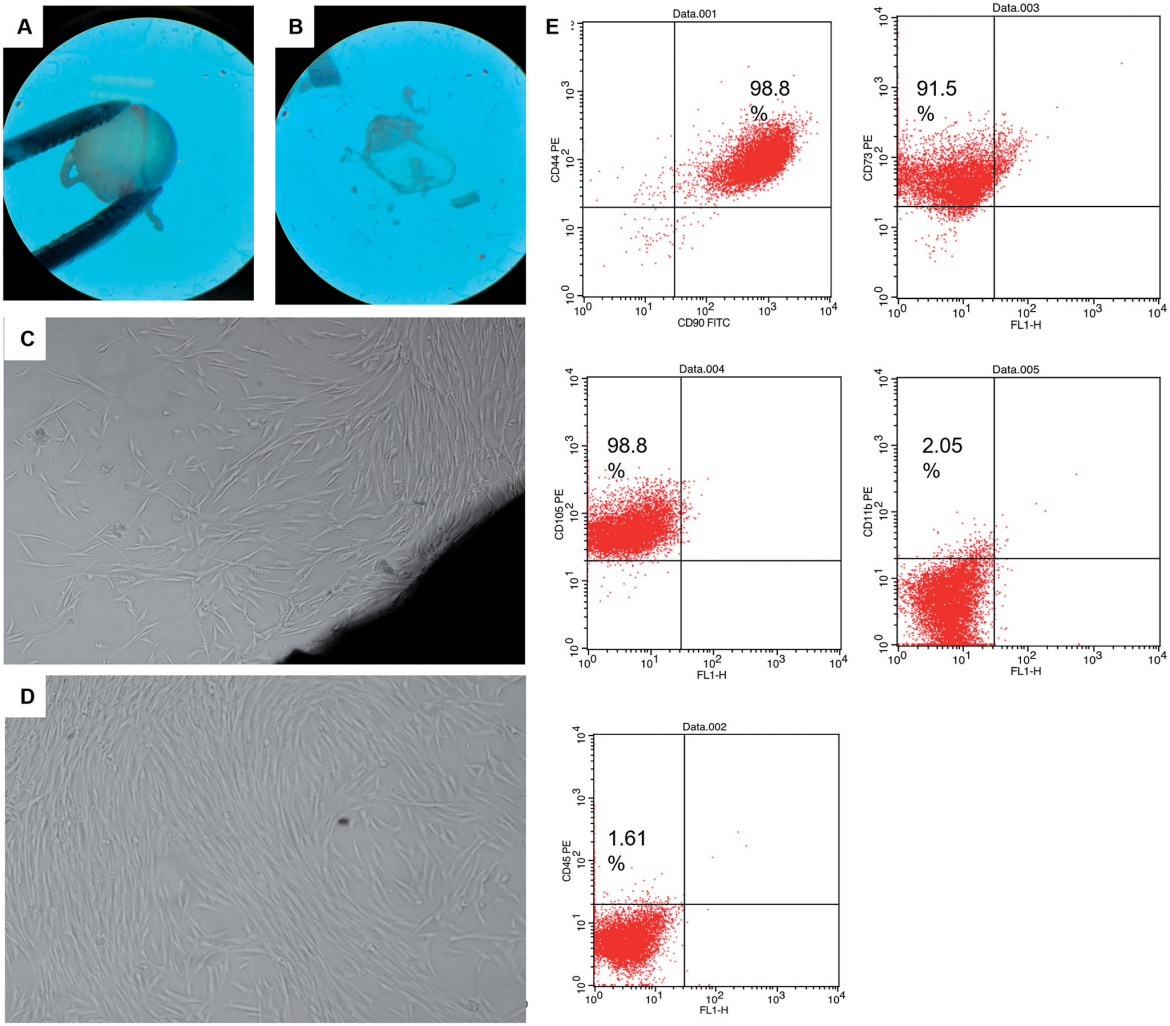
Corneal tissue dissection, culture and characterization: (A,B) dissected eyeballs and dissected corneal button visualized under stereomicroscope, (C) corneal explant showing corneal tissue in the lower right end and migrating spindle shaped cells from its margin, (D) passage 4 cultured spindle fibroblasts 90% confluent, and (E) scatter blots of fibroblasts characterization using CD surface markers CD90, CD44, 98.8%; CD73, 91.5%; CD105, 98.8%; CD11b, 2.05% and CD45, 1.61% using FACS caliber flow cytometer operated with Cell Quest software.

Corneal stromal fibroblasts were selected as a model for glaucomatous cells. Within 5–6 days of the corneal tissue explant culture, small spindle shaped cells were observed migrating out from the explants margins. The migrating cells possessed typical fibroblast like morphology and continued to proliferate till cells become confluent within another 7–9 days. Explants were then removed, and cells were trypsinzed and cultured in T75 for another three passages. Passage 4 cells’ characterization using surface markers revealed that 98.8% of cells express CD44 and CD 90, 91.5% express CD73 and 98.8% express CD105, while cells had very low expression of CD11b and CD 45 showing 2% and 1.6%, respectively (Figure 5).

#### 3.3.2. *In vitro* cytotoxicity assay

Instillation of topical eye drops directly contacts the surface of corneal cells, which might cause cytotoxic effects. For this reason, the ocular tolerability of the nanoparticle formulation should be taken into consideration before *in vivo* application. Fibroblasts served as a disease model for glaucomatous cells due to the fact that cells in the trabecular meshwork increasingly acquire the phenotype of contractile myofibroblasts during glaucoma development (Sonntag et al., 2021).

The cytotoxicity of free TET, TET-loaded BSA, and CS-coated BSA NPs was investigated on corneal stromal fibroblast cells by MTT assay. To test the potential cytotoxicity of NPs, corneal stromal fibroblast cells were treated with test samples at various concentrations (0.002, 0.02, 0.2, 2, 20 µg/mL). All samples tested did not show any cytotoxicity against cells in the tested concentration with cell viability above 95%, except for TET-BSA-NPs and CS-TET-BSA-NPs at 20 ug/mL where cell viability slightly decreased to 85% and 82%, respectively. However, all concentrations used showed no significant difference in cell viability with the control untreated cells in all formulations (Supplementary Figure 1). This is in agreement with previous studies reporting excellent cellular compatibility of albumin-NPs on different cell lines including RCE (Radwan et al., 2021) and ARPE-19 cells (de Redín et al., 2018).

#### 3.3.3. Cellular uptake studies of coumarin labeled BSA NPs

**Figure 6.**
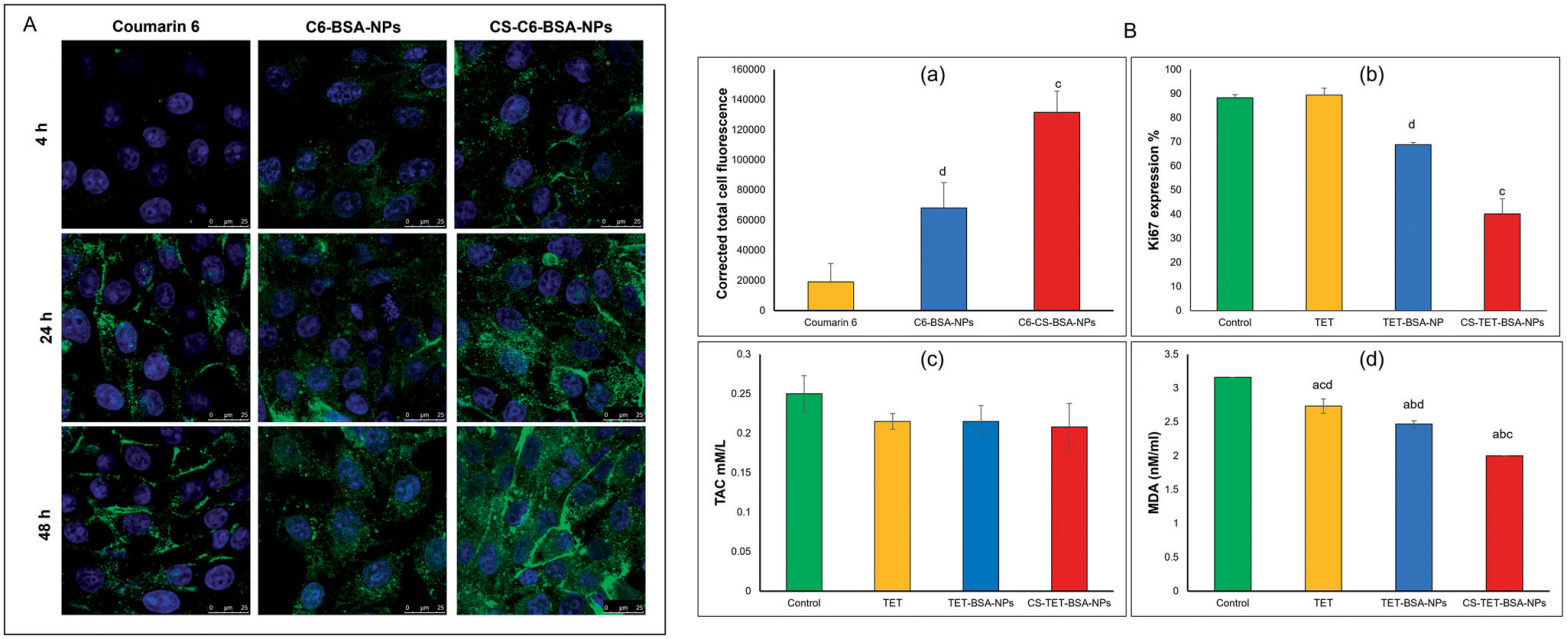
*In vitro* cell culture studies on corneal stromal fibroblasts: (A) confocal laser microscope images following incubation with TET formulations at different time intervals at 37 °C and (B) corrected total fluorescence following incubation for 48 h (*n* = 5) (a), antiproliferative activity expressed as %KI67 expression (b) and antioxidant effect expressed by total antioxidant capacity (TAC) and lipid peroxide (malondialdehyde, MDA), *n* = 3 (c and d). ^a^*p* < .05 versus control, ^b^*p* < .05 versus TET, ^c^*p* < .05 versus TET-BSA-NPs, ^d^*p* < .05 versus CS-TET-BSA-NPs.

The extent of coumarin labeled BSA-NPs and CS-BSA-NPs cell internalization by corneal stromal fibroblasts was evaluated by confocal microscopy. After 4 h exposure, free coumarin, which served as a control in this experiment, and negatively charged BSA NPs were internalized by the cells yet, the level of cellular uptake was significantly higher in case of the cationic CS coated BSA-NPs as shown in Figure 6(A,B-a). Fluorescence increased as the exposure time increased for the three groups indicative of increased cellular uptake. Cells treated with free coumarin showed the least fluorescence which significantly increased upon encapsulation into nanoparticles (*p* < .05). Also, NP formulations showed better distribution on corneal fibroblast cells.

CS-C6-BSA-NPs showed a significantly higher fluorescence intensity compared to C6-BSA NPs which could be explained by an enhancement in cellular uptake, this could be attributed to the positive charge of the CS coat.

#### 3.3.4. Anti-proliferative activity

A study conducted by Pitha et al., (2018) deduced that glaucoma causes a proliferative response as evidenced by increased expression of the proliferative marker KI67 (Oglesby et al., 2016). This response was present as early as 7 days after induction of ocular hypertension and remained significant for at least 6 weeks. Assessing fibroblast proliferation following IOP elevation could serve as a useful early marker for measuring the response to IOP elevation following medication treatment.

As illustrated in Figure 6(B-b), at the concentration tested, free TET had almost no antiproliferative effect on the corneal stromal fibroblasts with no significant difference (*p* > .05) compared to control cells, this could be a consequence of reduced cellular uptake. On the other hand, TET-BSA-NPs significantly (*p* ≤ .05) decreased the proliferative activity of KI67 when compared to TET and control cells. Further, a highly significant (*p* ≤ .01) reduction in proliferative activity was achieved by CS-TET-BSA-NPs (2.2- and 1.7-fold reduction compared to TET and TET-BSA-NPs, respectively). The superior antiproliferative potential of CS-TET-BSA-NPs is expected to synergize with IOP reducing potential and hence is relevant in the treatment of glaucoma (Pitha et al., 2018).

#### 3.3.5. Evaluation of oxidative stress

The oxidative injury and the altered antioxidant defense mechanisms observed in the pathophysiology of glaucomatous degeneration promoted the evaluation of systemic oxidative stress parameters and/or related antioxidants. Alteration in anti-oxidative biomarkers was observed in several clinical studies previously conducted on glaucoma patients (Cueto et al., 2021). In the current study, two biomarkers: total antioxidant capacity (TAC) and lipid peroxide (malondialdehyde, MDA) were selected to evaluate the antioxidant capacity of TET-BSA-NPs and CS-TET-BSA-NPs in comparison to free TET and control corneal stromal fibroblast cells (Figure 6(c,d)). Total antioxidant capacity (TAC) was almost unaltered (about 0.21 ± 0.04 mM/L) by all formulations tested with a statistically insignificant difference (*p* > .05) when compared to control cells (0.25 ± 0.02 mM/L). Nevertheless, MDA levels were decreased significantly (*p* ≤ .05) for corneal stromal fibroblasts that received both free TET (2.7 ± 0.105 nM/mL) and TET-BSA-NPs (2.47 ± 0.05 nM/mL) in comparison to the control group (3.16 ± 0 nM/mL). Further reduction in MDA level was achieved by CS-TET-BSA-NPs (2 ± 0.01 nM/mL, *p* < .01). in agreement with our results, Engin et al. (2010) reported that serum oxidative degradation products in glaucoma patients demonstrated an insignificant decrease in total antioxidant capacity (TAC) (Nath et al., 2017; Cueto et al., 2021), while MDA increased significantly indicating systemic lipid oxidation as a result of glaucoma.

### 3.4. Ocular tolerance test (HET-CAM test)

**Figure 7.**
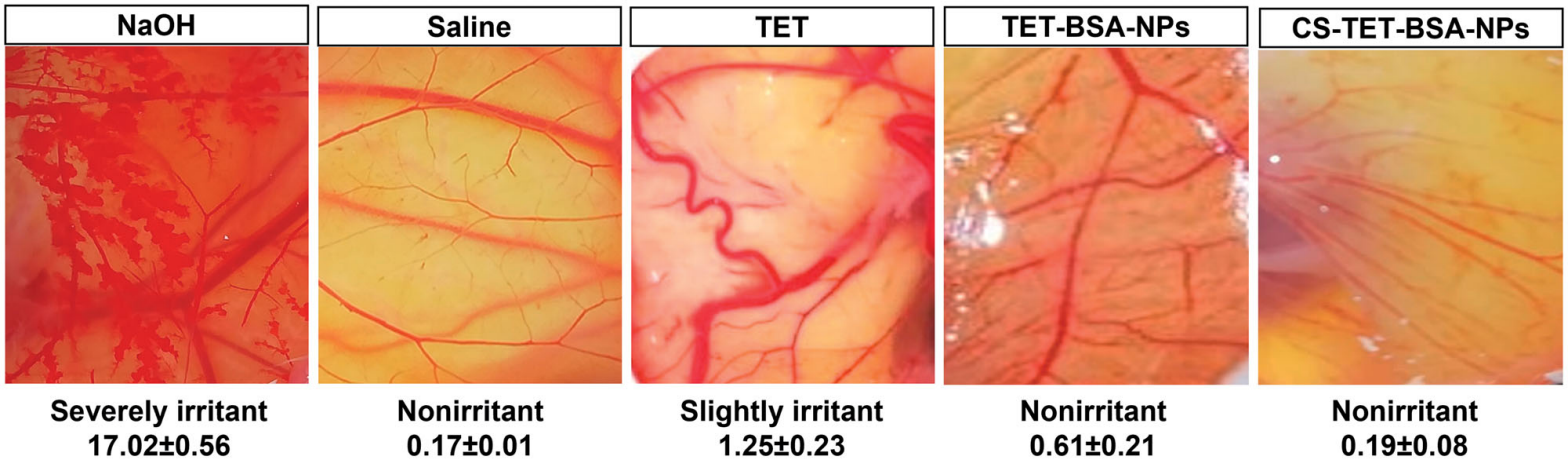
Photographs of hen’s egg test-chorioallantoic membrane (HET-CAM) after treatment at room temperature to predict ophthalmic irritation potential showing ocular irritation index value.

Hen's egg test-chorioallantoic membrane (HET-CAM) test was performed to qualitatively check ocular tolerability of TET suspension, TET-BSA-NPs, CS-TET-BSA-NPs relative to normal saline (negative control), and 0.1 N NaOH (positive control) (Figure 7). The scores were taken as per the OII after 5 min exposure. The mean OII of 0.1 NaOH (positive control)-treated group was 17.02 ± 0.56 indicating severe irritancy and significant damage to the blood vessels. On the other hand, the normal saline-treated group (negative control) showed no damage to the blood vessels with a mean OII 0.17 ± 0.01. Whereas TET solution OII was 1.25 ± 0.23 revealing a slightly irritant effect of the drug, a reduction in OII was obtained following TET loading into BSA-NPs (0.61 ± 0.21) and further decreased with CS coating (0.19 ± 0.08) which is statistically insignificant compared to the negative control group. This reflects the safety of the developed nano-formulations for ocular administration and their ability to reduce ocular irritancy of the drug making it more suitable for ocular drug delivery (Sánchez-López et al., 2018).

### 3.5. Aqueous humor pharmacokinetics

**Figure 8.**
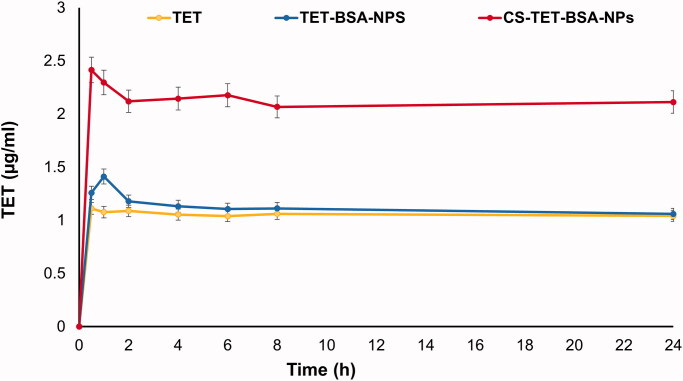
TET concentration-time profile in aqueous humor following topical application of TET, TET-BSA-NPs, and CS-TET-BSA-NPs to rabbits’ eyes (*n* = 3).

Aqueous humor TET concentration–time profiles upon topical instillation of TET suspension, TET-BSA-NPs, and CS-TET-BSA-NPs to the rabbit eye are shown in Figure 8. Calculated pharmacokinetic parameters are shown in Table 3. For the TET group, a relatively low ocular bioavailability (AUC_0–24_, 25.2 ± 10.4 µg·h/mL) was observed; the aqueous humor levels of the drug decreased and reached a plateau of 0.2 µg/mL after 1 h, which can be attributed to rapid pre-corneal clearance. Administration of TET-BSA-NPs yielded a slight insignificant (*p* > .05) increase in AUC_0–24_ (26.8± 11 µg·h/mL) compared to free TET suggesting the inability of BSA-NPs to penetrate the corneal barrier which could be a result of the negative charge of BSA-NPs. On the other hand, topical instillation of CS-TET-BSA-NPs showed significant enhancement in humoral TET availability where an almost 2-fold increase in *C*_max_ and AUC_0-24_ (51.2 ± 9.8 µg·h/mL) compared to TET and TET-BSA-NPs was observed. This could be attributed to increased corneal retention of the positively charged CS coat, providing a larger time frame for sustained release of the entrapped drug. Furthermore, the large surface area provided by nanosized particles in addition to mucoadhesiveness of CS increased the spreading and contact time over the corneal surface which strongly improved transcorneal TET permeation (Akhter et al., 2013). Similar findings were reported by Abu elkalam (Kalam, 2016) and Nagarwal et al. (2011) where high aqueous humor bioavailability suggested the mucoadhesiveness of CS-NPs and possible interaction with corneal and conjunctival epithelial layers of rabbit eyes, hence maintaining sufficiently high transcorneal concentration gradient. In addition, it was assumed that the positive surface of CS-NPs allows for interaction with the negative surface of the eye. Enhanced *ex vivo* transcorneal permeation and *in vitro* cellular uptake results further support the observed increase in bioavailability.

**Table 3.** Pharmacokinetic parameters following topical instillation of TET suspension, TET-BSA-NPs and CS-TET-BSA-NPs eye drops into rabbits’ eyes.

Pharmacokinetic parameter	TET suspension	TET-BSA-NPs	CS-TET-BSA-NPs
*C*_max_ (µg/mL)	1.11 ± 0.02	1.41 ± 0.4	2.41 ± 0.05
*T*_max_ (h)	0.5	1	0.5
AUC_0–24_ (µg·h/mL)	25.203 ± 10.4	26.802 ± 11	51.239 ± 9.8

### 3.6. IOP-lowering effect in rabbits

**Figure 9.**
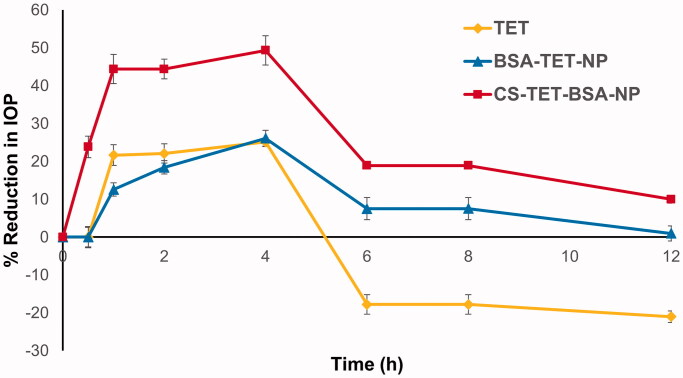
Percentage decrease in IOP after topical ocular instillation of a single dose of TET, TET-BSA-NPs, and CS-TET-BSA-NPs. Data expressed as mean ± SD (*n* = 3).

The pharmacological response (decrease in IOP, ΔIOP) versus time profiles for TET, TET-BSA-NPs, and CS-TET-BSA-NPs is presented in Figure 9. Success rate or effective therapy is defined as a minimum 15% IOP reduction from baseline (Akman et al. 2005), accordingly, all three formulations succeeded in lowering the IOP. TET suspension exhibited an IOP-lowering effect within 0.5–4 h with a maximum of 25.1 ± 3.8% at 4 h, and then, the effect wiped out, possibly due to the quick elimination of the drug from the corneal surface (Li et al., 2018). TET-BSA-NPs achieved a maximum IOP reduction of 26.1 ± 1.08% after 4 h which is not significantly different from TET suspension (*p* > .05). This is in agreement with the results of aqueous humor pharmacokinetics where the bioavailability of TET suspension and TET-BSA-NPs did not differ significantly. On the contrary, CS-TET-BSA-NPs showed a significantly enhanced reduction of the IOP when compared to TET solution and TET-BSA-NPs (*p* ≤ .05). A maximum IOP reduction of 49.35 ± 2.13% was observed for CS-TET-BSA-NPs after 4 h. This could be explained by CS interaction with mucin, facilitating the binding of the NPs with the corneal membrane and probably extending the absorption of TET, corneal permeation, and hence ocular bioavailability (Abdelmonem et al., 2021).

## 4. Conclusion

In this study, the influence of CS-coating on BSA NPs for ocular delivery was investigated. The coating with CS was confirmed by the change in ZP. CS coating resulted in drug release modulation and increased mucoadhesion of the NPs. Moreover, *ex vivo* transcorneal permeation on excised rabbit cornea showed significant enhancement by the CS coat. *In vitro* cell culture study results on corneal stromal fibroblasts reflected the safety and efficacy of the prepared formulations with superior cellular uptake, antiproliferative, and antioxidative capacities achieved following CS coating. HET-CAM egg irritation test confirmed the nontoxic nature of the NPs. Finally, CS-coated TET-BSA-NPs were able to significantly increase the pharmacokinetic parameters of TET enabling it lower IOP pressure in a rabbit animal model. Subsequently, the present research proposed a novel and promising strategy based on CS coating of BSA NPs for the topical delivery of TET to the anterior chamber of the eye to be used as an effective treatment of glaucoma with a low frequency of dosing and hence, better patient compliance
